# Supplementary material for: Comparative genomics provides new insights into the diversity, physiology, and sexuality of the only industrially exploited tremellomycete: Phaffia rhodozyma
Source: BMC Genomics. 2016 Nov 9;17:901. doi: 10.1186/s12864-016-3244-7 (PMC5103461; doi:10.1186/s12864-016-3244-7)
Supplement: Additional file 6: — List of orphan genes with links to PFAM (related to Additional file 1: Table S1). (ZIP 1428 kb) [file 12864_2016_3244_MOESM6_ESM.zip › PFAM_UNIQUE/G05849_P.html]

#                                                                            --- full sequence --- -------------- this domain -------------   hmm coord   ali coord   env coord
  
# target name        accession   tlen query name           accession   qlen   E-value  score  bias   #  of  c-Evalue  i-Evalue  score  bias  from    to  from    to  from    to  acc description of target
  
#------------------- ---------- ----- -------------------- ---------- ----- --------- ------ ----- --- --- --------- --------- ------ ----- ----- ----- ----- ----- ----- ----- ---- ---------------------
  
G05849\_P             -            103 RNF111\_N             PF15303.1    269       3.9    5.6   7.4   1   2      0.42   6.3e+02   -1.7   0.1   188   198    20    30     6    45 0.47 - E3 ubiquitin-protein ligase Arkadia N-terminus  
G05849\_P             -            103 RNF111\_N             PF15303.1    269       3.9    5.6   7.4   2   2   0.00048      0.72    8.0   3.7   225   255    68    98    60   101 0.90 - E3 ubiquitin-protein ligase Arkadia N-terminus
